# Complete mitochondrial genome of the psammophilic fungus *Mallocybe* sp. (Inocybaceae, Basidiomycota) from Cambodia

**DOI:** 10.1128/mra.00360-25

**Published:** 2025-06-10

**Authors:** Amal F. Alsaqaf, Ana C. Avila Armenta, Lupita Avila, Caedence Barclay, Azucena D. Bautista, Yenethsy Bernal, Joshua D. Betts, Jacob D. Cain, Jesus Camargo, Celso De Jesus, Jonathan Esquivel-Zavala, Alexa Flores, Yuvia Francisco, Timothy L. Fuller, Lizbeth E. Galvez, Jade Gonzalez Cruz, Jesus Gonzalez, Angel Gutierrez, Virgilio Hernandez Cruz, Julissa M. Hernandez, Marisa M. Hertzog, Jeffery R. Hughey, Sandy Huynh, Shemar M. Joseph, Jasleen Kaur, Leah Lopez, Alexis Luquin-Sanchez, Britania Mandujano, Jocelynn Martin, Yasmin Martinez, Yolanda Martinez, Brianna M. Meskus, Gloria Meza, Emmah Migotti, Elida J. Mosqueda, Marisol Nambo, Emily Osorio, Krisha J. Panchal, Gabriel C. Panlilio, Jema Ramirez-Gonzalez, Alondra Reyes-Ayala, Diego Rodriguez, Karina Rodriguez, Maria I. Russo, Andrea Salazar, Alvaro E. Sanchez Lopez, Maria Suarez, Sarah N. Takemura, Angela M. Tavarez, Ulyses Urias Rivas, Shea Valenzuela, SeoJung Yoon, On behalf of Hartnell College Genomics Group

**Affiliations:** 1 Division of Mathematics, Science, and Engineering, Hartnell College 17023, Salinas, California, USA; University of Maryland School of Medicine, Baltimore, Maryland, USA

**Keywords:** Agaricales, bioinformatics, *Casuarina equisetifolia*, high-throughput sequencing, mitogenome

## Abstract

We present the complete mitochondrial genome of a psammophilic *Mallocybe* sp. from Koh Rong Sanloem, Cambodia. The mitochondrial genome of *Mallocybe* sp. is circular, AT skewed (70.2%), and 21,500 bp in length. It contains the conserved fungal gene package, including 16 protein-coding genes, 23 transfer RNA, and 2 ribosomal RNA genes.

## ANNOUNCEMENT

*Mallocybe* (Kuyper) Matheny, Vizzini & Esteve-Rav. is a basidioma-forming fungal genus with widespread global distribution and 81 legitimate species names (1–5). Its species typically inhabit soil in mixed forests and have ectomycorrhizal associations with a wide range of plants (1, 6). *Mallocybe* is characterized by its woolly and yellowish-brown basidioma, short cheilocystidia, and reaction with ammonium hydroxide (1, 2). To date, there are many published phylogenetic investigations on species of *Mallocybe* based on nuclear markers (6–9); however, no mitochondrial genomes have been deciphered. To contribute to the bioinformatics and systematics of the Inocybaceae, the complete mitochondrial genome of an undescribed species of *Mallocybe* from Cambodia was assembled and characterized.

**Fig 1:**
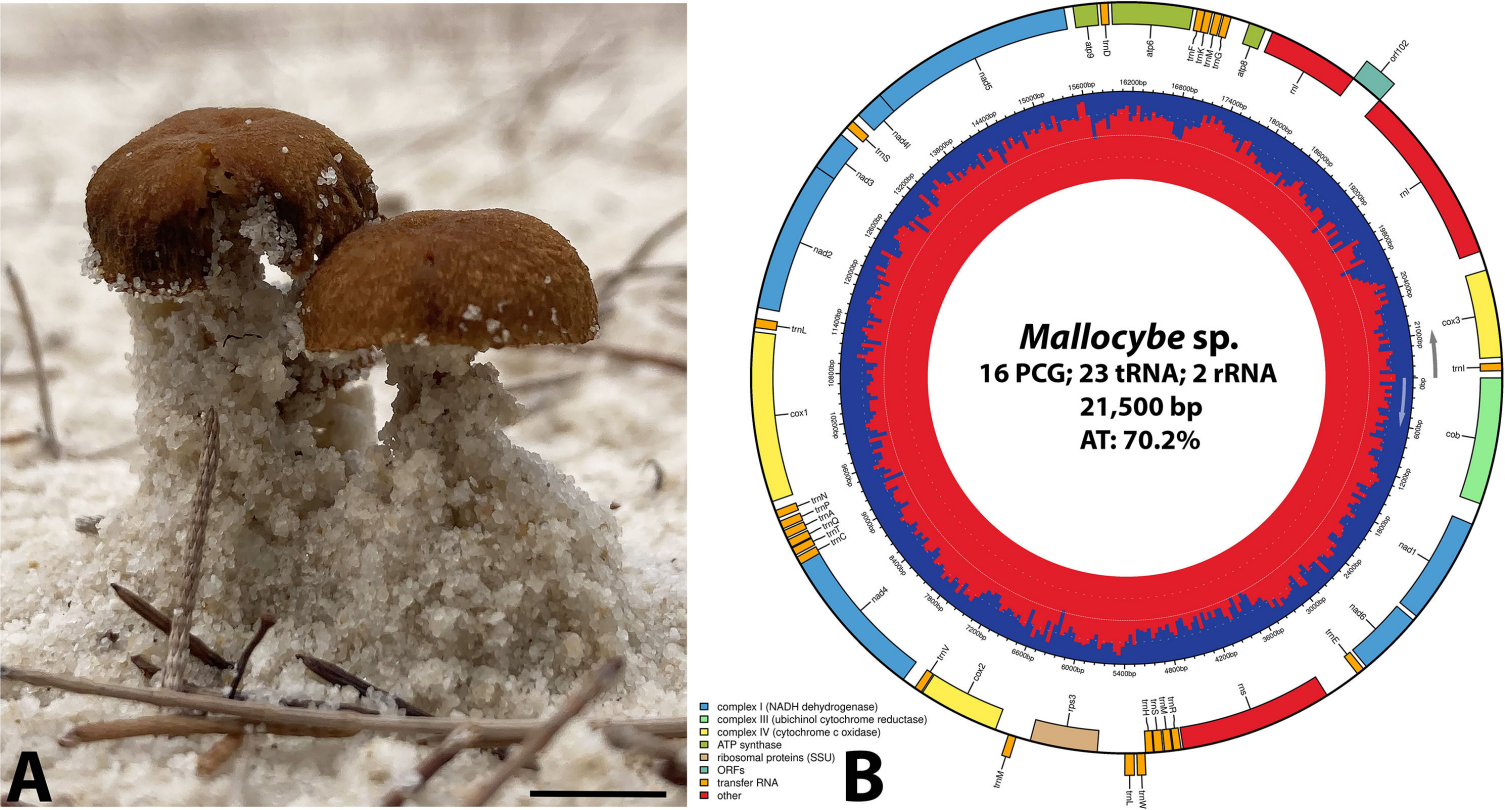
*Mallocybe* sp. basidioma habit (**A**) and the complete mitochondrial genome map (**B**). (**A**) Specimens of *Mallocybe* sp. from Koh Rong Sanloem, Cambodia. Scale bar = 5 mm. (**B**) Mitochondrial genome map of *Mallocybe* sp. PCG, protein-coding genes. The genome was mapped with Chloroplot 0.2.4 (19). The innermost ring displays the AT content in red color and the direction of transcription, as indicated by the arrows. The final ring displays the genes. Genes transcribed clockwise are on the inside, while counterclockwise transcriptions are positioned on the outside. The color coding corresponds to genes of different groups as listed in the key at the bottom left.

The specimen of *Mallocybe* sp. was collected just above the high-water mark on a sandy beach, growing in association with *Casuarina equisetifolia* L., on Koh Rong Sanloem, Cambodia (10°35′00.5″N 103°18′18.3″E), voucher number: Hartnell College Collection #274 (Fig. 1A). The DNA was extracted from the silica gel-dried pileus using the DNeasy Blood and Tissue Kit (Qiagen) following the manufacturer’s protocol with two modifications: the binding step was at 4,000 *g* for 3 minutes, and the DNA was eluted in 40 µL TAE after 7 minutes of incubation (10). The 150 bp paired-end library was constructed with the NEBNext Ultra II DNA Library Prep Kit (New England BioLabs) and sequenced on an Illumina NovaSeq 6000 (Illumina, Inc.) by Novogene Corporation Inc. The sequencing generated 13,295,496 reads that were filtered using the default BBDuk 1.0 (11) settings in Geneious Prime 2019.1.3 (Biomatters Limited). The mitochondrial genome was assembled using the trimmed reads with GetOrganelle 1.7.5+ with the default parameters, MEGAHIT 1.2.9 with kmers 79, 99, 119, and 141, and NOVOPlasty 4.3.1 with a *Mallocybe terrigena* seed (GenBank accession number FJ904183) and default settings (12–14). All three assemblers recovered a single, circular, identical mitochondrial genome with an average coverage of 6,727×. The annotation was predicted with MFannot version 3.0 (15) using the default mode and manually adjusting start and stop positions according to NCBI ORF finder (https://www.ncbi.nlm.nih.gov/orffinder/), StructRNAfinder 1.0 (16), tRNAscan-SE 2.0 (17), and Sequin 15.5 (18). The internal transcribed spacer (ITS) sequence was assembled by mapping the cleaned reads of *Mallocybe* sp. to *Mallocybe sabulosa* (Matheny & Bougher) Matheny & Esteve-Rav. (GenBank accession number KP308821) using the Medium-Low Sensitivity/Fast setting with three iterations in Geneious Prime.

The complete circular mitochondrial genome of *Mallocybe* sp. is 21,500 bp in length and has an AT% skew of 70.2% (Fig. 1B). The assembled mitochondrial genome falls within the range (13,032–343,690 bp) of published basidiomycete mitochondrial genomes (20). The organellar *Mallocybe* sp. genome contains the standard core genes, including 16 protein-coding genes (PCGs), 23 tRNA, and 2 rRNA genes (Table 1) (20, 21). Fifteen of the PCGs initiate with the ATG codon, and one (cox2) with TTG. Most of the PCGs terminate with TAA; however, three (atp6, nad4, and orf102) share the stop codon TAG (Table 1). The ITS sequence of *Mallocybe* sp. from Cambodia (GenBank accession number PV461560) is identical in sequence to *Mallocybe* sp. (GenBank accession number OP204693) from China (9).

**TABLE 1:** Mitochondrial genome content, organization, and codon information of *Mallocybe* sp. (GenBank accession number PV364444)^*a*^

Gene	Type	Minimum nucleotide position	Maximum nucleotide position	Length	Start codon	Stop Codon	Direction
cob	CDS	1	1,170	1,170	ATG	TAA	Forward
nad1	CDS	1,372	2,355	984	ATG	TAA	Forward
nad6	CDS	2,402	3,004	603	ATG	TAA	Forward
trnE-UUC	tRNA	3,038	3,108	71	–	–	Forward
rns	rRNA	3,448	4,856	1,409	–	–	Forward
trnR-UCU	tRNA	4,876	4,946	71	–	–	Forward
trnM1-CAU	tRNA	4,957	5,028	72	–	–	Forward
trnS1-GCU	tRNA	5,042	5,123	82	–	–	Forward
trnH-GUG	tRNA	5,131	5,202	72	–	–	Forward
trnW-CCA	tRNA	5,209	5,282	74	–	–	Reverse
trnL1-UAA	tRNA	5,308	5,391	84	–	–	Reverse
rps3	CDS	5,644	6,264	621	ATG	TAA	Forward
trnM2-CAU	tRNA	6,397	6,469	73	–	–	Reverse
cox2	CDS	6,597	7,343	747	TTG	TAA	Forward
trnV-UAC	tRNA	7,356	7,426	71	–	–	Forward
nad4	CDS	7,556	9,025	1,470	ATG	TAG	Forward
trnC-GCA	tRNA	8,973	9,046	74	–	–	Forward
trnT-UGU	tRNA	9,068	9,139	72	-–	–	Forward
trnQ-UUG	tRNA	9,150	9,223	74	–	–	Forward
trnA-UGC	tRNA	9,244	9,315	72	–	-–	Forward
trnP-UGG	tRNA	9,334	9,407	74	–	–	Forward
trnN-GUU	tRNA	9,441	9,513	73	–	–	Forward
cox1	CDS	9,600	11,171	1,572	ATG	TAA	Forward
trnL2-UAG	tRNA	11,211	11,294	84	–	–	Forward
nad2	CDS	11,396	12,793	1,398	ATG	TAA	Forward
nad3	CDS	12,794	13,165	372	ATG	TAA	Forward
trnS2-UGA	tRNA	13,246	13,326	81	–	–	Forward
nad4l	CDS	13,403	13,669	267	ATG	TAA	Forward
nad5	CDS	13,669	15,549	1,881	ATG	TAA	Forward
atp9	CDS	15,633	15,854	222	ATG	TAA	Forward
trnD-GUC	tRNA	15,883	15,955	73	–	–	Forward
atp6	CDS	15,987	16,736	750	ATG	TAG	Forward
trnF-GAA	tRNA	16,773	16,844	72	–	–	Forward
trnK-UUU	tRNA	16,845	16,914	70	–	–	Forward
trnM3-CAU	tRNA	16,936	17,005	70	–	–	Forward
trnG-UCC	tRNA	17,021	17,091	71	–	–	Forward
atp8	CDS	17,267	17,422	156	ATG	TAA	Forward
rnl	rRNA	17,493	20,324	2,523	–	–	Forward
orf102	CDS	18,345	18,653	309	ATG	TAG	Reverse
cox3	CDS	20,503	21,312	810	ATG	TAA	Forward
trnI-GAU	tRNA	21,365	21,435	71	–	–	Forward

^*a*^ “–,” not applicable.

## Data Availability

The complete mitochondrial genome sequence of *Mallocybe* sp. is available in GenBank under accession number PV364444. The associated BioProject, SRA, and BioSample numbers are PRJNA1244861, SRS24560579, and SAMN47732872, respectively.

## References

[1] Matheny PB, Hobbs AM, Esteve-Raventós F. 2020. Genera of Inocybaceae: new skin for the old ceremony. Mycologia 112:83–120. doi:10.1080/00275514.2019.1668906 31846596

[2] Liu F, Liu C, Chen X-H, Chen X-H, Lan Z-Y, Xu X-L, Liu Y-G, Yang C-L. 2024. A novel Mallocybe species (Inocybaceae, Agaricales) discovered in the Longquan Mountain of Southwestern China. Phytotaxa 659:56–66. doi:10.11646/phytotaxa.659.1.4

[3] Robert Vincent, Vu D, Amor ABH, van de Wiele N, Brouwer C, Jabas B, Szoke S, Dridi A, Triki M, Ben Daoud S, et al.. 2013. MycoBank gearing up for new horizons. IMA Fungus 4:371–379. doi:10.5598/imafungus.2013.04.02.16 24563843 PMC3905949

[4] Crous PW, Gams W, Stalpers JA, Robert V, Stegehuis G. 2004. MycoBank: an online initiative to launch mycology into the 21st century. Stud Mycol 50:19–22.

[5] Robert V, Stegehuis G, Stalpers J. 2005. The mycoBank engine and related databases. Available from: https://www.MycoBank.org

[6] Matheny PB, Kudzma LV, Graddy MG, Mardini SM, Noffsinger CR, Swenie RA, Walker NC, Campagna SR, Halling R, Lebeuf R, Kuo M, Lewis DP, Smith ME, Tabassum M, Trudell SA, Vauras J. 2023. A phylogeny for North American Mallocybe (Inocybaceae) and taxonomic revision of eastern North American taxa. Fungal Syst Evol 12:153–201. doi:10.3114/fuse.2023.12.09 38455953 PMC10918758

[7] Matheny PB. 2005. Improving phylogenetic inference of mushrooms with RPB1 and RPB2 nucleotide sequences (Inocybe; Agaricales). Mol Phylogenet Evol 35:1–20. doi:10.1016/j.ympev.2004.11.014 15737578

[8] Aignon HL, Naseer A, Matheny BP, Yorou NS, Ryberg M. 2021. Mallocybe africana (Inocybaceae, Fungi), the first species of Mallocybe described from Africa. Phytotaxa 478:49–60. doi:10.11646/phytotaxa.478.1.3

[9] Hu JH, Yu WJ, Deng LS, Fan YG, Bau T, Tang LP, Lin WF, Deng CY. 2023. The detection of major clades and new species of Mallocybe (Inocybaceae, Agaricales) from China with elongate cheilocystidia. Mycol Progress 22:15. doi:10.1007/s11557-022-01854-5

[10] Garcia AN, Ramos JH, Mendoza AG, Muhrram A, Vidauri JM, Hughey JR, Hartnell College Genomics Group. 2022. Complete chloroplast genome of topotype material of the coast live oak Quercus agrifolia Née var. agrifolia (Fagaceae) from California. Microbiol Resour Announc 11:e0000422. doi:10.1128/mra.00004-22 35254126 PMC9022551

[11] Bushnell B. 2014. BBMap: a fast, accurate, splice-aware aligner. United States. https://www.osti.gov/biblio/1241166.

[12] Jin J-J, Yu W-B, Yang J-B, Song Y, dePamphilis CW, Yi T-S, Li D-Z. 2020. GetOrganelle: a fast and versatile toolkit for accurate de novo assembly of organelle genomes. Genome Biol 21:241. doi:10.1186/s13059-020-02154-5 32912315 PMC7488116

[13] Li D, Liu CM, Luo R, Sadakane K, Lam TW. 2015. MEGAHIT: an ultra-fast single-node solution for large and complex metagenomics assembly via succinct de Bruijn graph. Bioinformatics 31:1674–1676. doi:10.1093/bioinformatics/btv033 25609793

[14] Dierckxsens N, Mardulyn P, Smits G. 2016. NOVOPlasty: de novo assembly of organelle genomes from whole genome data. Nucleic Acids Res 45:gkw955. doi:10.1093/nar/gkw955 PMC5389512 28204566

[15] Lang BF, Beck N, Prince S, Sarrasin M, Rioux P, Burger G. 2023. Mitochondrial genome annotation with MFannot: a critical analysis of gene identification and gene model prediction. Front Plant Sci 14:1222186. doi:10.3389/fpls.2023.1222186 37469769 PMC10352661

[16] Arias-Carrasco R, Vásquez-Morán Y, Nakaya HI, Maracaja-Coutinho V. 2018. StructRNAfinder: an automated pipeline and web server for RNA families prediction. BMC Bioinformatics 19:55. doi:10.1186/s12859-018-2052-2 29454313 PMC5816368

[17] Chan PP, Lin BY, Mak AJ, Lowe TM. 2021. tRNAscan-SE 2.0: improved detection and functional classification of transfer RNA genes. Nucleic Acids Res 49:9077–9096. doi:10.1093/nar/gkab688 34417604 PMC8450103

[18] Benson DA, Cavanaugh M, Clark K, Karsch-Mizrachi I, Ostell J, Pruitt KD, Sayers EW. 2018. GenBank. Nucleic Acids Res 46:D41–D47. doi:10.1093/nar/gkx1094 29140468 PMC5753231

[19] Zheng S, Poczai P, Hyvönen J, Tang J, Amiryousefi A. 2020. Chloroplot: an online program for the versatile plotting of organelle genomes. Front Genet 11:576124. doi:10.3389/fgene.2020.576124 33101394 PMC7545089

[20] Tang J, Zhang L, Su J, Ye Q, Li Y, Liu D, Cui H, Zhang Y, Ye Z. 2024. Insights into fungal mitochondrial genomes and inheritance based on current findings from yeast-like fungi. JoF 10:441. doi:10.3390/jof10070441 39057326 PMC11277600

[21] Kouvelis VN, Kortsinoglou AM, James TY. 2023. The evolution of mitochondrial genomes in fungi, p 65–90. In Pöggeler S, James T (ed), Evolution of fungi and fungal-like organisms. Springer, Berlin/Heidelberg, Germany.

